# Prospective assessment of the ability of rapid shallow breathing index computed during a pressure support spontaneous breathing trial to predict extubation failure in ICU

**DOI:** 10.1186/cc14312

**Published:** 2015-03-16

**Authors:** G Besch, J Revelly, P Jolliet, L Piquilloud-Imboden

**Affiliations:** 1 CHRU Besançon, France; 2 CHUV, Lausanne, Switzerland

## Introduction

As the objective clinical criteria [1] are imperfect to assess patients before extubation, simple physiological parameters are used to try to improve extubation failure (EF) prediction. The rapid shallow breathing index (RSBI) (respiratory rate (RR) over tidal volume (VT) ratio) recorded during a T-piece spontaneous breathing trial (SBT) is known as the most reliable physiologic predictor. However, RSBI is nowadays usually computed during a pressure support (PS) SBT using the values displayed on the ventilator screen and not based on spirometry measurements without any assist as initially published. The aim of the present study was to prospectively assess the ability of currently measured RSBI to predict EF.

## Methods

Retrospective analysis of prospectively collected data from patients intubated for more than 48 hours admitted in the medico-surgical ICU of Lausanne, Switzerland, from January 2007 to December 2008. EF was defined as the need for reintubation within 48 hours after extubation. Reintubations for a procedure requiring general anesthesia were not considered as EFs. RR and VT during the PS SBT were recorded from the ventilator and RSBI was computed accordingly. Baseline characteristics and currently measured RSBI were compared between patients who experienced EF versus success (*t *test or chi-square test as appropriate). The ability of currently measured RSBI to predict EF was assessed using ROC curve analysis.

## Results

A total of 478 extubated patients were included, 25 of whom (5.2%) were reintubated. ICU mortality (ICU-m) and in-hospital mortality (H-m) were higher in reintubated patients: ICU-m = 6 (24) versus 22 (5), *P *= 0.002 and H-m = 9 (36) versus 63 (15), *P *= 0.009. The reasons for EF were: acute lung failure (*n *= 15), congestive heart failure (*n *= 4) and aspiration/bronchial congestion (*n *= 6). Demographic data were similar between patients successfully and nonsuccessfully extubated: age: 58 ± 17 versus 58 ± 19 years, *P *= 0.85; male gender: 15 (60) versus 263 (61), *P *= 0.99. SAPS II score was higher in the EF group: 30 ± 22 versus 42 ± 27, *P *= 0.04. RSBI were significantly higher in patients who experienced EF: RSBI = 59 ± 44 versus 43 ± 26, *P *= 0.04. The area under the ROC curve for currently measured RSBI was: 0.617 (95% CI = 0.571 to 0.662), *P *= 0.035.

## Conclusion

In a cohort of 458 medico-surgical ICU patients, RSBI measured during a pressure support SBT was higher in patients experiencing EF but very imperfect to predict EF.
